# OPNa Overexpression Is Associated with Matrix Calcification in Thyroid Cancer Cell Lines

**DOI:** 10.3390/ijms19102990

**Published:** 2018-09-30

**Authors:** Luciana B. Ferreira, Raquel T. Lima, Ana Clara Santos da Fonseca Bastos, Andreia M. Silva, Catarina Tavares, Ana Pestana, Elisabete Rios, Catarina Eloy, Manuel Sobrinho-Simões, Etel R. P. Gimba, Paula Soares

**Affiliations:** 1 i3S-Instituto de Investigação e Inovação em Saúde, Universidade do Porto, 4200-135 Porto, Portugal; luciana.bueno.ferreira@gmail.com (L.B.F.); rlima@ipatimup.pt (R.T.L.); andreia.silva@ineb.up.pt (A.M.S.); ctavares@ipatimup.pt (C.T.); apestana@ipatimup.pt (A.P.); erios@ipatimup.pt (E.R.); ssimoes@ipatimup.pt (M.S.-S.); 2 Institute of Molecular Pathology and Immunology of the University of Porto (Ipatimup), 4200-135 Porto, Portugal; celoy@ipatimup.pt; 3 Research Coordination, National Institute of Cancer, Rio de Janeiro 20230-130, Brazil; anaclarafonseca_93@hotmail.com; 4 Medical Faculty, University of Porto, 4200-319 Porto, Portugal; 5 INEB—Instituto de Engenharia Biomédica, 4200-135 Porto, Portugal; 6 ICBAS—Instituto de Ciências Biomédicas Abel Salazar da Universidade do Porto, 4050-313 Porto, Portugal; 7 Department of Pathology, Hospital de S. João, 4200-319 Porto, Portugal; 8 Natural Sciences Department, Health and Humanities Institute, Fluminense Federal University, Rio de Janeiro 28880-000, Brazil

**Keywords:** osteopontin, papillary thyroid carcinoma, psammoma bodies, matrix calcification, OPNa

## Abstract

Osteopontin (OPN) spliced variants (OPN-SV: OPNa, OPNb, and OPNc) are aberrantly expressed in tumors and frequently associated with cancer progression. This holds true for papillary thyroid carcinoma (PTC), which is the most common type of thyroid cancer (TC). PTC often presents with desmoplasia and dystrophic calcification, including psammoma bodies (PB). This work aimed to investigate total OPN (tOPN) and OPN-SV expression and their association with the presence of PB in the PTC classical variants (cPTC), as well as the involvement of OPN-SV in matrix calcification of TC cell lines. We found that cPTC samples presenting PB showed higher OPN expression levels. In TC cell lines, OPNa overexpression promotes higher matrix calcification and collagen synthesis when compared to that of clones overexpressing OPNb or OPNc. In response to OPN knockdown, calcification was inhibited, paralleled with the downregulation of calcification markers. In conclusion, our data evidenced that OPN expression is associated with the presence of PB in cPTC samples. Among the OPN-SV, OPNa is the main contributor to matrix calcification in tested TC cells, providing clues to a better understanding on the biology and ethiopathogenesis of the calcification process in TC cells.

## 1. Introduction

Thyroid cancer (TC) is the most prevalent endocrine malignancy, and papillary thyroid carcinoma (PTC) the most common histologic type, accounting for approximately 80–90% of human TCs [1,2]. The growing incidence of TC is almost entirely attributed to the increased incidence of PTC cases [2,3]. PTC displays genetic alterations, such as *RET/PTC* rearrangements, *BRAF^V600E^*, and *RAS* mutations. However, the etiopathogenesis of these tumors is not completely understood [4].

Previous reports have shown that clinicopathological features, such as patient age, sex, tumor size, histological subtype, extrathyroid extension, and lymph node status are useful prognostic factors in PTC patients [5,6,7]. Calcification is a frequent histological feature in several cancers and has been usually detected by ultrasonography at preoperative assessment of thyroid nodules [8,9]. Despite matrix calcification not being exclusive of malignant thyroid lesions, its association with malignancy has been previously described [10,11].

Based on histological features, TC calcification may consist in dystrophic calcification or in the formation of psammoma bodies (PB) [12]. PB are defined as spherical (50–70 μm in diameter, calcified foci with concentric laminations, presenting a glassy appearance [13,14,15]. The genesis of PB in PTC is not completely understood. Some authors have proposed that PB may be formed by (i) vascular stalk of the neoplastic papillae, starting with a basal lamina thickening, followed by vascular thrombosis, calcification, and tumor cell necrosis and/or (ii) necrosis and calcification in intralymphatic tumor thrombi at the thyroid tissues adjacent to tumor or in the opposite thyroid lobe [14]. The presence of PB is easily detected in cytological or histological specimens. In fine-needle aspiration (FNA) biopsies, the presence of PB has been correlated to malignancy, and it is a *bona fide* marker of PTC diagnosis [10]. In PTC, PB are almost exclusively observed in classical variants of PTC (cPTC) and also in some rare PTC variants, such as diffuse sclerosing variant (dsPTC) [16].

Several molecules contribute to the calcification process, such as osteopontin (OPN), collagen type I and osteocalcin [17]. Collagen type I is an important component of the bone extracellular matrix (ECM), forming connections with cell surface integrins and other ECM proteins [17]. Osteocalcin is one of the most abundant proteins present in bone, second only to collagen type I [18]. It is thought to play an important role in osteoblast progenitor cell differentiation, with significant up-regulation in both matrix synthesis and calcification [19]. OPN is recognized as a multifunctional phosphoglycoprotein, being involved in bone remodeling and mineralization. In non-tumoral bone tissues, OPN is expressed by osteoclasts and osteoblasts, which are the cells responsible for bone remodeling. Notably, OPN has calcium-binding properties and hydroxyapatite affinity. It has been proposed that the phosphate groups’ steric arrangements at the OPN structure are required for calcium binding and apatite crystal formation during bone matrix mineralization [20].

OPN primary transcript suffers alternative splicing, generating at least three variants, named OPNa, OPNb, and OPNc, which perform tissue and tumor specific roles [21].

Earlier reports showed that total OPN (tOPN) overexpression (which corresponds to the sum of all OPN variants) seems to play a role on the formation of PB in PTC samples. Tunio et al. [22] observed that OPN overexpression in PTC cells was found around PB and that OPN expressing cells were identified as CD68-positive macrophages. In a more recent study, OPN expression in PTC samples was also significantly associated with the presence of PB [23].

Recently, we demonstrated that OPNa (which corresponds to the full length coding sequence) is expressed in higher levels than OPNb and OPNc in cPTC samples. Our group previously described that OPNa overexpression was associated with PTC aggressive clinicopathological features. Additionally, we showed that OPNa overexpression induced cell proliferation, migration, motility, and invasion in TC cell lines [24].

Despite the aforementioned studies, current knowledge is scarce regarding molecular interactions that result from PB in cPTC and OPN overexpression, especially considering the relative contribution of each OPN spliced variant (OPN-SV) in these processes. The goal of this study was to evaluate the putative associations between the expression of tOPN and its splice variants with the presence of PB. As an approach to better understand the putative correlations between OPN expression and their splice variants and cPTC tissue calcification process, we also investigated the involvement of OPN expression on matrix calcification and collagen synthesis in TC cell lines.

## 2. Results

### 2.1. cPTC Samples Containing PB Exhibit Higher tOPN Staining Intensity

**Figure 1:**
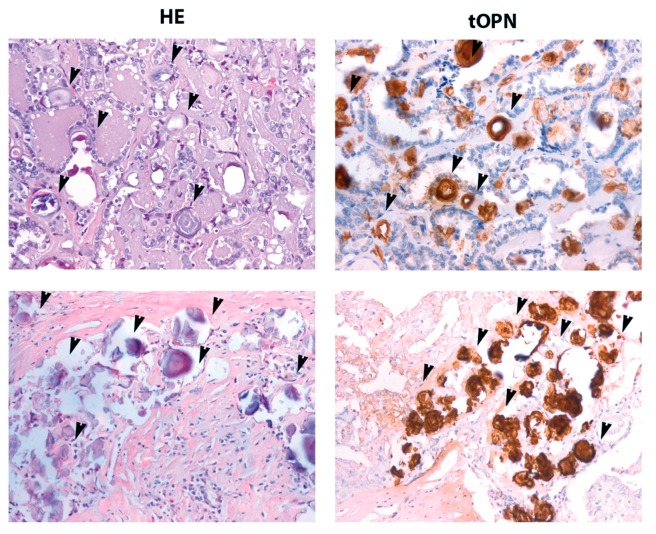
Total Osteopontin (OPN) staining at psammoma bodies (PB) in classical variants of papillary thyroid carcinoma cPTC cases. Two different representative cPTC cases (**upper** and **lower panel**) showing psammoma bodies with black arrow heads. **Left panel**: PB stained by HE, 40×. **Right panel**: PB stained for total OPN (tOPN) antibody, 40×.

Immunohistochemistry (IHC) staining for tOPN was performed in 48 samples from cPTC tissues. A total OPN staining score was established according to our previous report [25]. Total OPN expression was observed in 60.4% of the cPTC samples (29 out of 48) (Table S1). HE stained samples were carefully analyzed for the presence of PB. As shown in Figure 1, right panel, PB were strongly stained by tOPN antibody, allowing a much clearer view of PB in cPTC samples, when compared to haematoxylin and eosin (HE) staining approach.

According to anti-tOPN antibody staining, PB were detected in 53% (24 out of 45) of cPTC samples. High protein staining scores of tOPN were associated with the presence of stroma in cPTC samples (*p* = 0.01) (Table S2). No statistically significant association was found between the presence of PB and tOPN expression, although cPTC samples containing PB presented higher tOPN staining intensity (tOPN score mean: 2.54 vs. 2.29). Similarly, no association was found between tOPN protein expression and sex, age, tumor size, extrathyroid extension, lymphovascular and/or capsular invasion, lymph node metastases, *RET/PTC* rearrangements, *BRAF^V600E^*, or *RAS* mutations. We then evaluated the association between the presence of PB and some clinicopathological and molecular features of cPTCs. We found a significant association between the presence of PB and younger patient age (*p* = 0.005) and with the presence of lymph node metastases (*p* = 0.03) (Table S3). No significant associations were found between the occurrence of PB and patients’ sex, presence of stroma, tumor size, extrathyroid extension, lymphovascular and/or capsular invasion, *RET/PTC* translocation, *BRAF^V600E^*, and *RAS* mutations.

A regression model was performed to evaluate factors associated with the presence of lymph node metastases in cPTCs (Table S4). A total of 14 patients (40%) had lymph node metastases. Younger age (odds ratio (OR) 4.4; *p* = 0.05) and the presence of PB (OR 7.3; *p* = 0.02) were associated with the presence of lymph node metastases. However, in the multivariate regression analysis, younger patient age and the presence of PB were not significantly associated with the presence of lymph node metastases.

### 2.2. OPNa Transcript Overexpression Is Associated with Presence of PB in cPTC Samples

Since we found a higher tOPN protein expression in cPTC samples containing PB (although not attaining statistical significance), we then evaluated transcript expression levels of tOPN and each OPN-SV (OPNa, OPNb, or OPNc) in order to analyze their relative contributions to PB formation. Of note, tOPN expression corresponds to the sum of all OPN variants, including post-translational, splicing and alternative translation initiation variants. In this study, we found that cPTC samples containing PB also have higher tOPN, OPNa, OPNb, and OPNc transcript levels, in relation to those samples in which PB are lacking. Interestingly, only OPNa and OPNb levels were significantly higher (*p* < 0.05) in samples containing PB (Figure S1 and Table 1).

### 2.3. OPNa Overexpression Induces Matrix Calcification in c643 Thyroid Cells

**Figure 2:**
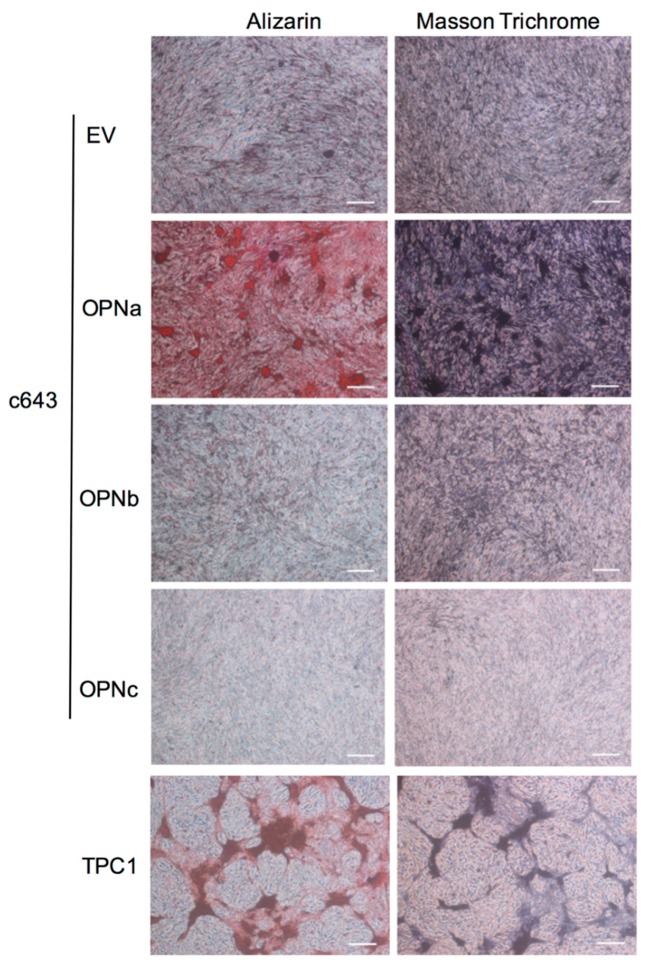
Calcification and collagen production in c643 cell clones and TPC-1 cell line. **Left panel**: c643 cell clones overexpressing OPNa, OPNb, or OPNc, as well empty (EV) controls were analyzed (**Left panel**) for matrix calcification by Alizarin Red staining, in which darker orange areas represent extracellular matrix (ECM) areas rich in calcium deposits. **Right panel**: c643 cell clones and TPC-1 cells were analyzed for collagen deposits by Masson trichrome staining. Dark purple areas correspond to extracellular matrix rich in collagen. Scale bar: 100 µM. Representative photomicrographs of two independent experiments at 24 days of cell culture are shown.

The ability of c643 thyroid cells overexpressing each OPN-SV to produce calcified extracellular matrix (ECM) was evaluated by Alizarin Red staining following 24 days in cell culture (Figure 2).

We found that c643 cells overexpressing the OPNa variant presented higher Alizarin Red staining intensity when compared to that of cell clones overexpressing OPNb or OPNc variants. These results showed that OPNa-overexpressing cells were able to produce higher levels of calcified ECM (Figure 2, left panel).

### 2.4. OPNa Overexpression Promotes Collagen Synthesis in c643 Thyroid Cancer Cell Line

Since PB formation is described to rely on matrix calcification, in association with some additional ECM components, such as collagen [26], we then analyzed whether the three OPN-SV differently contribute to induce collagen synthesis in c643 cells. For this purpose, c643 OPN-SV overexpressing cells were cultured for 24 days and then stained with Masson trichrome. We observed that OPNa overexpression induced higher collagen synthesis when compared with that of OPNb or OPNc overexpressing clones, as shown by the Masson trichrome dark purple-red staining (Figure 2, right panel).

### 2.5. TPC-1 Thyroid Cancer Cell Line Produce Matrix Calcification and Collagen Synthesis

Since we found in our previous data that among nine TC cell lines analyzed for OPN expression [24], TPC-1 presented the highest endogenous OPNa variant expression levels, we decided to analyze if this cell line was also able to promote matrix calcification and synthesize collagen. Our data revealed that TPC-1 cells, in basal conditions, produced calcification and collagen synthesis in the ECM, even without the ectopic OPN-SV overexpression (Figure 2, lower panel).

### 2.6. OPNa and Calcification Markers Are Upregulated in TPC-1 Cell Line during Culture Time Course

**Figure 3:**
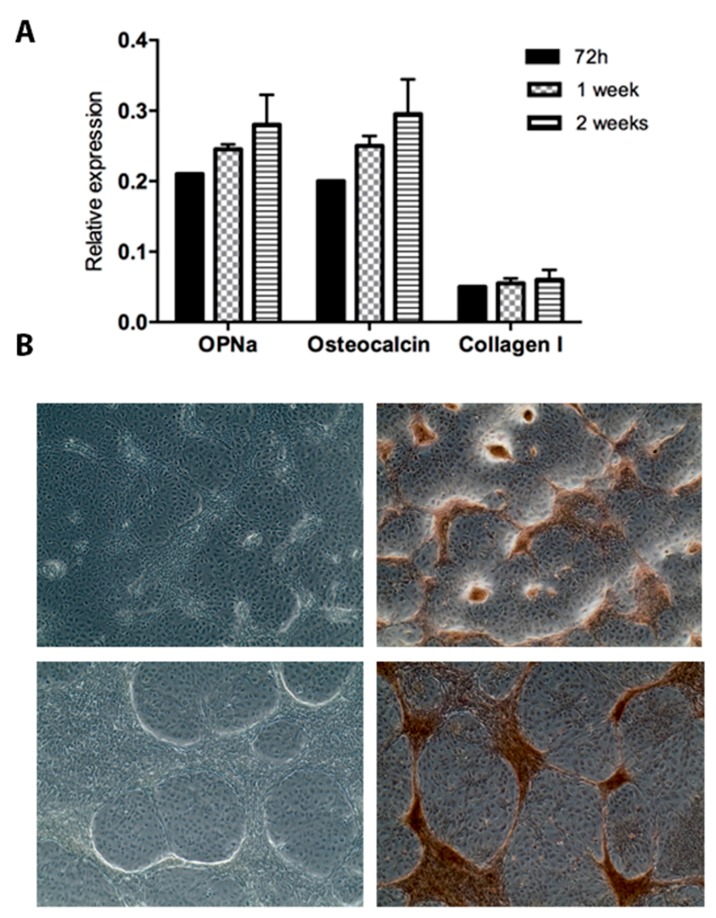
OPNa, osteocalcin, and collagen type I transcript levels and calcification deposits in TPC-1 cell line. (**A**) OPNa, osteocalcin, and collagen type I messenger RNA (mRNA) expression levels was measured by RT-qPCR in the TPC-1 thyroid cell line after 72 h, 1 week, and 2 weeks of cell culture. (**B**) Representative phase contrast images for 72 h (**upper panel**) and 1 week (**lower panel**) of cell culture of TPC-1 cell line are shown in the left column. In the right column, matrix calcification was detected with Alizarin Red staining after 72 h and 1 week of cell culture. Dark brown areas correspond to ECM rich in calcium deposits.

Based on data observed for TPC-1 cells regarding high matrix calcification, we then evaluated the expression of OPNa and calcification markers such as osteocalcin and collagen type I during a cell culture time course. We found that the transcriptional levels of calcification markers, as well as OPNa, were upregulated after 72 h, one week, and two weeks of cell culture (Figure 3A).

Additionally, at the 72-h and 1-week time points, strong matrix calcification was also observed in TPC-1 cells (Figure 3B).

### 2.7. OPN Silencing Decreases Matrix Calcification and the Expression of Calcification Markers in TPC-1 Cell Line

**Figure 4:**
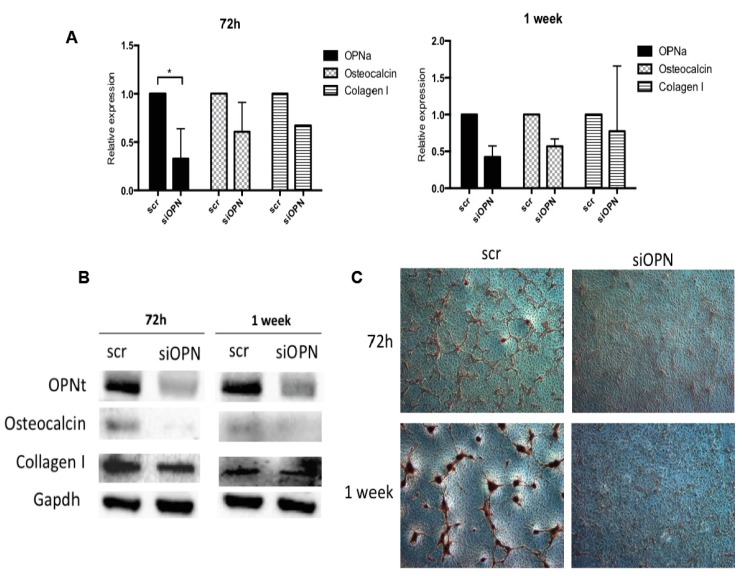
OPN silencing in TPC-1 cells promotes downregulation of OPNa, osteocalcin, collagen type 1 mRNA, and protein expression and inhibition calcification deposits. (**A**) OPNa, osteocalcin, and collagen type I mRNA expression levels were measured by real time PCR in TPC-1 thyroid cell line after 72 h and 1 week of cell culture. Cells were treated with 100 nM of small interfering RNA (siRNA) for tOPN and 100 nM of siRNA negative control (scr), corresponding to scr-siRNA, for 72 h and 1 week. (**B**) Western blot was performed for OPN, osteocalcin, and collagen type I proteins after OPN silencing. Representative GAPDH expression is shown. The protein levels in treated cells were evaluated in duplicate. (**C**) Representative images 72 h after OPN silencing in TPC-1 cell line and for the siRNA negative control (scr,) are shown. Matrix calcification was detected with Alizarin Red staining after 72 h and 1 week of OPN silencing and the siRNA negative control (scr). Dark brown areas correspond to ECM rich in calcium deposits.

To further investigate whether the upregulated expression of osteocalcin and collagen type 1 are modulated by OPN expression levels, we knocked down tOPN expression in TPC-1 cell line using an OPN-small interfering RNA (OPN-siRNA). A scrambled (scr)-siRNA sequence was used as a negative control. At 72 h post-transfection with OPN-siRNA, the transcript levels of OPNa, osteocalcin, and collagen type I were significantly downregulated in TPC-1 cells (Figure 4A, left panel).

OPNa and osteocalcin transcript levels remained downregulated one week after OPN-siRNA transfection. OPNb and OPNc expression levels were also diminished in response to OPN silencing (Figure S2). Otherwise, collagen type I transcript levels increased in this time point (Figure 4A, right panel). We also analyzed the protein expression levels of these calcification markers by immunoblot. We found that tOPN, osteocalcin, and collagen type I protein expression levels were decreased at 72 h and 1 week post-transfection (Figure 4B). Matrix calcification was also evaluated in TPC-1 cells in which OPN has been knocked down. One week after OPN silencing in TPC-1 cells, matrix calcification was reduced, when compared to that of scr-siRNA transfected cells (Figure 4C). These results indicate that OPN expression levels regulate osteocalcin and collagen type I expression in TPC-1 cells, possibly contributing to the matrix calcification process.

## 3. Discussion

In this study, we investigated the expression of the matricellular protein OPN and its splice variants in cPTC samples and their relation with the presence of PB. Moreover, we also investigated the putative contributions of each OPN-SV on the matrix calcification in TC cell lines. We found that cPTC tumor samples presenting PB are associated with younger patient age and the presence of lymph node metastases. We also found that higher OPNa transcript levels are associated with the presence of PB in cPTC samples. Noteworthy, OPNa variant overexpression strongly induced matrix calcification and collagen deposition and contributed to upregulated expression of *bona fide* calcification markers, such as collagen type I and osteocalcin in TC cell lines.

Our results also indicate that PB can be more clearly defined in PTC samples stained for tOPN than using HE staining, since PB are strongly stained for anti-OPN antibody (Figure 1). To date, only two studies reported associations between OPN expression and the occurrence of PB in PTC samples [22,23]. Another association between OPN and cell matrix calcification in cancer has been reported by Hirota et al. [27], who described OPN protein co-localization with calcium phosphate deposits in meningioma tissues, further evidencing a role for OPN on the PB formation in this context.

We further showed that cPTC samples that presented PB also had higher expression levels of tOPN, OPNa, OPNb, and OPNc transcripts than that of samples in which PB was lacking. Notably, OPNa expression is significantly higher than OPNb transcript levels in these samples. In a previous work, we demonstrated that among the three OPN-SV, OPNa had the highest expression levels in cPTC samples when compared with that of other thyroid tissues [24]. Therefore, the current results are in accordance with our earlier observations, raising a putative association between OPNa variant overexpression and the formation of PB in cPTC.

Regarding the analysis of cPTC clinicopathological features, we found a significant correlation between cPTC cases presenting PB and the presence of lymph node metastases and with younger patients. PB is a diagnostic indicator for cPTC, and its presence strongly suggests tumor malignancy in preoperative diagnosis. Bai et al. [13] also reported that the presence of PB in PTC cases was associated with gross lymph node metastases and high-stage TC tumor (stage IVa). Conversely, Pyo et al. [28] found an association between PB and tumor multifocality, extrathyroid extension, and lymph node metastases. Higher expression of OPNa in cPTC samples containing PB in relation to those samples in which PB are absent and the association of PB with lymph node metastases is in accordance with our previous data, in which we showed that OPNa overexpression seems to contribute to cPTC progression [24]. Bai et al. [13] also found an association between stromal calcification and advanced patient age (>60 years). However, these authors only considered stromal calcification, which frequently arises in benign lesions, whereas PB are suggestive of malignancy. Herein, in our cPTC samples, only PB have been observed, excluding other dystrophic or stromal calcifications and bone formation. In fact, no association was found between OPNa transcript levels and dystrophic calcification. In our previous work, we also reported an association between tOPN protein expression and the presence of stroma in cPTC cases, suggesting that this may be correlated with tumor aggressiveness in cPTCs [24]. In order to establish putative impact of OPN-SV on the matrix calcification process, OPNa, OPNb, and OPNc were overexpressed in c643 TC cells [24]. We provided in vitro evidence of an association between OPNa expression and the deposition of calcium and collagen at the ECM.

Our data demonstrated that among the three OPN-SV, OPNa is the major activator of matrix calcification. Conversely, OPN has been mainly described as an inhibitor of calcification, such as in bones [29,30] and vascular calcification [29]. However, OPN has also been previously reported as an activator of calcification, such as in dental pulp [31,32] and craniopharyngioma calcification [33]. We then proposed that in the thyroid tumor cell context, OPN may behave as a positive modulator of matrix calcification, as has been proposed by others, in which OPN may be involved in the control of calcification rather than its genesis [34]. Several reports revealed that calcification is more common in malignant than in benign thyroid nodules [35,36]. Other authors reported that intrathyroidal calcification was noted in 26.1% (29 out of 111) of malignant thyroid nodules and in only 8.0% (20 out of 250) of benign thyroid samples [37]. Nonetheless, it is important to mention that many authors have been stressing that the presence of intra-thyroid calcification *per se* cannot be used to distinguish between benign and malignant thyroid disease [38,39], although the presence of PB is a strong indicator of PTC presence.

We also observed that c643 cells overexpressing OPNa induced higher collagen synthesis in the ECM. These results are in accordance with our previous observations, whereby OPNa appeared to be the key OPN variant associated with the presence of stroma [24].

These data have been validated in TPC-1 cells, which endogenously express high OPN levels [24]. Similar to what has been observed for c643 cells overexpressing OPNa, TPC-1 cells also exhibited calcium deposits and production of collagen fibers. The TPC-1 cell line is recognized as presenting the *RET/PTC* rearrangements [40]. This molecular alteration was demonstrated to induce OPN expression in thyroid cell lines, such as PCCl3 [41], reinforcing the hypothesis that OPN plays a role in such processes.

To further demonstrate the involvement of OPN in the matrix calcification process of cPTC cells, we found that OPN knockdown in TPC-1 cells could regulate the expression of collagen type I and osteocalcin calcification markers. The downregulation of these calcification markers in response to OPN knockdown indicated that OPN can also contribute to the calcification process in TPC-1 thyroid cancer cell line. Although our results provide evidence that OPNa may have a more prominent role in such a mechanism, further studies should better investigate how OPNa modulates collagen synthesis and the calcification process. OPN silencing approach used in this work was not specific to OPNa, but could rather target any OPN-SV. Once in TPC-1 cell lines, OPNa is the major OPN-SV, we expected that most silenced OPN variants should correspond to OPNa. Nevertheless, we found that OPNb and OPNc levels were also decreased. We hypothesize that this could be a result of siRNA targeting these three OPN-SV and/or the effect of OPNa levels on modulating the expression of the other two splice variants. Moreover, considering that c643 and TPC-1 cell lines are representative of anaplastic thyroid carcinoma [42], future studies should validate OPNa roles on promoting matrix mineralization and collagen synthesis in PTC cell line models overexpressing each OPN-SV.

Remarkably, OPNa’s differential roles on modulating matrix mineralization and collagen synthesis is possibly correlated to its related structural differences when compared to OPNb and OPNc variants. For instance, some authors have discussed that phosphorylation of OPN is an important factor in regulating the OPN-mediated mineralization process [43,44]. The structural differences among these three OPN splicing isoforms relates to the lack of exon 4 and exon 5 in OPNc and OPNb isoforms, respectively. These exons are rich in phosphorylation sites, and deletion of these exons in OPNc and OPNb variants changes OPN phosphorylation patterns [45]. It has also been proposed that conservation of phosphorylation sequences in OPN has been correlated to fundamental roles in many of its biological functions, including bone remodeling [46]. Additionally, it has been shown that some of OPN’s effects over osteoclast roles require OPN phosphorylation [47]. Additionally, osteoblasts at distinct differentiation stages are able to alter OPN post-translational modifications (PTMs), including phosphorylation and sulphation [48,49]. Therefore, based on these previous findings, in addition to our current data, we propose that OPNa, which contains the whole OPN sequence and related functional domains preserving all the phosphorylation sites, can better contribute not only to several pro-tumorigenic roles in thyroid cancer cells [24], but also to the matrix calcification process.

In conclusion, our data showed that OPNa variant expression levels is associated with the occurrence of PB in cPTC samples and is able to more efficiently promote matrix calcification and collagen synthesis, as well as to promote the expression of calcification markers in TC cells in vitro. These data support an important role for OPNa on matrix calcification in these TC tested cell lines, providing new clues to a better understanding of cPTC biology and its etiopathogenesis.

## 4. Materials and Methods

### 4.1. Tumor Specimens

Tissue specimens were collected from primary tumors, surgically resected at the Centro Hospitalar São João (CHSJ) at Porto, Portugal. This tumor series is composed of 48 cases of cPTC. After surgery, samples were immediately snap-frozen and stored at −80 °C until use. Additional fragments were fixed in 10% buffered formalin and embedded in paraffin (FFPE). The histologic diagnosis of all cases was reviewed by specialized pathologists, according to the World Health Organization (WHO) classification criteria [50]. Clinicopathological and molecular features of patients and corresponding tumors are summarized in Table 2. All the procedures described in this study are in accordance with national and institutional ethical standards. This work was approved by the Ethic Committee for Health (CES) of the Hospital Center of São João (CHSJ) (Porto, Portugal)/Faculty of Medicine of the University of Porto (FMUP) (Porto, Portugal) (CES 137 284-13). All the procedures described in this study were in accordance with national ethical standards (Law no 12/2005) and Helsinki declaration. Patients signed an informed consent form.

### 4.2. Cell Culture

The expression levels of tOPN and OPN-SV were established in TC cell lines in a previous study from our group [24]. For this study, the TPC-1 and c643 cell lines (presenting the highest and the lowest tOPN and OPN-SV levels, respectively) were selected for experimental approaches.

We used c643 cells stably overexpressing OPN-SV, as described in Ferreira LB et al. [24]. Briefly, the open reading frame of OPNa, OPNb, and OPNc were cloned into pCR3.1 mammalian expression vector as previously described by He B et al. [51]. These plasmid constructs were used to transfect c643 cells using Lipofectamine 2000 (Invitrogen, Carlsbad, CA, USA). The OPN splice variants/pCR3.1 plasmids or the expression vectors that do not contain the cloned circular DNA (cDNA) (Empty Vector) were transfected into c643 cells and the stably overexpressing cells were selected with 600 μg/mL of G418 in the culture medium. All the cell lines were cultured in Roswell Park Memorial Institute (RPMI) 1640 cell culture medium with glutamine, supplemented with 10% fetal bovine serum (FBS), 100 IU/mL penicillin, and 100 mg/mL streptomycin in a humidified environment containing 5% CO_2_ at 37 °C.

### 4.3. Immunohistochemistry

OPN immunohistochemistry (IHC) analysis was performed in representative tumor tissue sections using an anti-total OPN antibody (anti-tOPN) (polyclonal, goat, 1:500, R & D Systems, Minneapolis, MN, USA) which recognizes all three OPN-SV. Normal gallbladder tissue samples previously reported to overexpress tOPN [25] was used as a positive control. IHC procedures were done according to Ferreira LB et al. [25]. Semi-quantitative IHC evaluation was independently performed by two observers (CE and LBF). Total OPN staining was scored in the range 0–7, corresponding to the sum of the staining intensity (absent = 0, faint = 1, moderate = 2 and strong = 3) plus the proportion of positively stained cells (<5% = 0; 5–25% = 1; 25–50% = 2; 50–75% = 3; and >75% = 4).

### 4.4. Matrix Calcification Assessment

In order to evaluate matrix calcification, c643 stably overexpressing OPN-SV and TPC-1 cell lines were cultured in 24-well plates (1.5 × 10^5^ cells/well) for 31 days. Following 10, 17, 24, or 31 days in culture, cells were fixed with 4% paraformaldehyde (PFA) in PBS and evaluated for calcium deposits production by staining with 1% Alizarin Red solution in 2% ethanol. Cells were then washed three times with water, and representative images for each culture condition were captured with a NIKON microscope and NIKON Digital DLS Camera.

### 4.5. Matrix Collagen Synthesis Assay

We cultured c643 overexpressing OPN-SV and TPC-1 cell lines in 24-well plates (1.5 × 10^5^ cells/well) for 31 days. Following 10, 17, 24, or 31 days in culture, cells were fixed with 4% paraformaldehyde (PFA) in PBS and evaluated for collagen fibers formation by Masson’s trichrome staining. Briefly, cells were immersed in Richard-Allan Scientific^®^ Bouin’s fluid (Thermo Fisher Scientific, Waltham, MA, USA) solution for 5 min at room temperature, washed in deionized water, followed by incubation in Celestin Blue (Thermo Scientific) for 6 min. Then, cells were incubated in Gil’s hematoxylin staining solution (Sigma-Aldrich, St. Louis, MO, USA) for 5 min. Cells were washed with 1% acid alcohol and then underwent three sequential washes in deionized water. Cells were then immersed in Biebrich scarlet-acid fuchsin (Thermo Scientific) for 5 min, placed in phosphotungstic and phosphomolybdic acid solution (Thermo Scientific) for 5 min, moved to Aniline Blue solution (Thermo Scientific) for 5 min, and then placed in 1% acetic acid solution for 2 min. Finally, cells were rinsed in deionized water. Representative images for each culture condition were captured with a NIKON microscope and NIKON Digital DLS Camera.

### 4.6. OPN Silencing

When TPC-1 cell cultures reached around 70–80% confluence, they were transfected with specific OPN-siRNA (Cat#AM16708A; Ambion INC.) and a scramble (scr)-siRNA negative control (Cat#AM4611; Ambion INC.) using Lipofectamine™ 2000 liposome transfection kit (cat. no. 11668027; Thermo Fisher Scientific, Inc., Waltham, MA, USA). After 6 h, RPMI 1640 medium containing 10% serum was added, and the cells were incubated for 72 h or one week in a 5% CO_2_ incubator. Quantitative reverse transcription PCR (RT-qPCR) and immunoblot assays were used to evaluate, respectively, transcript and protein expression of OPN, osteocalcin, and collagen I, as described below.

### 4.7. RNA Extraction and RT-qPCR

Total RNA was extracted from cell lines and tumor tissues using Trizol reagent (Life Technologies, Carlsbad, CA, USA). For cDNA preparation, 1 μg of total RNA was reverse transcribed using the RevertAid first-strand cDNA synthesis kit (Invitrogen, Carlsbad, CA, USA). qPCR assays were carried out for each OPN-SV transcript (OPNa, OPNb, and OPNc) using the SYBR Green detection system (Applied Biosystems, Warrington WA1 4SR, UK). Amplifications were performed in a 10 μL reaction volume containing 200 μg of cDNA, 0.2 μM of each primer (forward and reverse), and 5 μL of SYBR Green mix. The oligonucleotide primers are listed in Supplementary Table S5. The following amplification conditions were used: 50 °C for 2 min, 94 °C for 5 min followed by 40 cycles of 94 °C for 30 s, 60 °C for 30 s, and 72 °C for 45 s. Relative transcript expression was calculated using the Delta-Delta CT method. *GAPDH* gene was used as the housekeeping control. The melting curve was analyzed, ramping from 65 °C to 95 °C (increment 0.5 °C/5 s) in order to verify the amplicon specificity based on the presence of a single and sharp peak.

### 4.8. Immunoblot Analysis

TPC-1 cells transfected with OPN-siRNA or the scr-siRNA were lysed in RIPA lysis buffer supplemented with phosphatase and protease inhibitors. Proteins were quantified using DCTM Protein Assay (Bio-RAD, Hercules, CA, USA) and then resolved by SDS-PAGE and transferred onto nitrocellulose membranes (GE Healthcare, Little Chalfont, UK). The primary antibodies were anti-tOPN (O-17 #18625) (1:500) from IBL Immuno-Biological Laboratories; anti-collagen type I—sc-8784 (1:400), and anti-osteocalcin sc-30044 (1:800) from Santa Cruz Biotechnology, Santa Cruz, CA, USA. Protein was detected using a horseradish peroxidase-conjugated antibody (Santa Cruz Biotechnology, Santa Cruz, CA, USA) and a luminescence system (Perkin-Elmer, Waltham, MA, USA). For protein loading control, membranes were incubated with an anti-GAPDH antibody from Santa Cruz Biotechnology (Santa Cruz, CA, USA). Protein expression was quantified using the Bio-Rad Quantity One 1-D Analysis software (Bio-Rad Laboratories, Inc., Hercules, CA, USA). All proteins were normalized by GAPDH loading control.

### 4.9. Statistical Analysis

Statistical analysis was performed using version 22.0 SPSS statistical package (IBM, 2014). Independent-sample *t*-test was performed to verify if there were any associations between OPN expression and the presence of PB. The significance of any associations between the presence of PB and the clinicopathological features was determined by either the *X*^2^ test or Fisher’s exact test (two-sided). The predictive value of PB and other factors (sex, age, presence of stroma, tumor size, absence of capsule, extrathyroid extension, invasion, lymph node metastases, *RET/PTC* rearrangement, *BRAF^V600E^*, and *RAS* mutations) were assessed using univariate and multivariate logistic regression models. For in vitro experiments, data are presented as mean ± standard error and were analyzed using Mann-Whitney test. Values of *p* < 0.05 were considered statistically significant.

## Figures and Tables

**Table 1:** Summary of the clinical, pathological, and molecular data of the cPTC cases.

Variable	cPTC *n* (%)
Sex *	
Male	6 (15)
Female	41 (85)
Age (yr) (mean ± S.D.) *	39.87 (±16.42)
<45	26 (56.5)
≥45	20 (43.4)
Stroma	
Absent	21 (44)
Present	27 (56)
Tumor size (cm) *	
(mean ± S.D.)	2.4 (±1.1)
<2	17 (37)
≥2	29 (63)
Capsule*	
Absent	24 (56)
Present	19 (44)
Psammoma Bodies *	
Absent	21 (47)
Present	24 (53)
Extrathyroid Extension *	
Absent	24 (57)
Present	18 (43)
Invasion Lympho (vascular and/or capsular) *	
Absent	15 (35)
Present	28 (65)
Lymph Node Metastases *	
Absent	21 (60)
Present	14 (40)
*RET/PTC1* translocation *	
Absent	37 (84)
Present	7 (16)
*BRAF^V600E^* mutation *	
Absent	22 (48)
Present	24 (52)
*RAS* mutation *	
Absent	44 (96)
Present	2 (4)

* Clinicopathological data were missing in some cases included in the series.

**Table 2:** Association between tOPN and OPN- spliced variant (OPN-SV) transcript expression levels with the presence of PB in cPTC samples.

	Psammoma Bodies		
Variable	Absent (*n* = 19)	Present (*n* = 26)	*p*-Value
tOPN mRNA expression (Median)	0.15	0.18	0.73
OPNa mRNA expression (Median)	0.12	0.14	**0.01**
OPNb mRNA expression (Median)	0.10	0.11	**0.04**
OPNc mRNA expression (Median)	0.05	0.05	0.07

## Supplementary Materials

The following are available online at http://www.mdpi.com/1422-0067/19/10/2990/s1, Figure S1: Expression levels of OPNa, OPNb, OPNc and tOPN transcripts in cPTC samples, concerning presence or absence of PB. (A) OPNa (B) OPNb (C) OPNc and (D) tOPN mRNA expression levels were measured by real <me PCR in the cPTC samples. * *p* < 0.05. Results are from at least two independent assays with triplicates. Figure S2: Expression levels of three OPN splice variants in response to total OPN silencing; Table S1: Staining score of tOPN IHC in cPTC samples; Table S2: Correlation between tOPN protein expression and the clinicopathological characteristics in cPTC; Table S3: Correlation between the presence of PB and the clinicopathological characteristics in cPTC; Table S4: Predictive factors for lymph node metastases in papillary thyroid carcinomas; Table S5: Forward and reverse oligonucleotide sequences.
