# Supplementary material for: OPNa Overexpression Is Associated with Matrix Calcification in Thyroid Cancer Cell Lines
Source: Int J Mol Sci. 2018 Sep 30;19(10):2990. doi: 10.3390/ijms19102990 (PMC6213506; doi:10.3390/ijms19102990)
Supplement: Supplementary file 1 [file ijms-19-02990-s001.zip › ijms-356325-SI.pdf]

International Journal of *Molecular Sciences*

MDPI

Supplementary Material

OPNa Overexpression Is Associated with Matrix Calcification in Thyroid Cancer Cell Lines

Luciana B. Ferreira [1,2,3], Raquel T. Lima [1,2,4], Ana Clara Santos da Fonseca Bastos [3], Andreia M. Silva [1,5,6], Catarina Tavares [1,2], Ana Pestana [1,2], Elisabete Rios [1,2,4,7], Catarina Eloy [2], Manuel Sobrinho-Simões [1,2,4,7], Etel R. P. Gimba [3,8,]* and Paula Soares [1,2,4,]*

[1] i3S-Instituto de Investigacão e Inovacão em Saúde, Universidade do Porto, 4200-135 Porto Portugal; luciana.bueno.ferreira@gmail.com (L.B.F.); rlima@ipatimup.pt (R.T.L.); andreia.silva@ineb.up.pt (A.M.S.); ctavares@ipatimup.pt (C.T.); apestana@ipatimup.pt (A.P.); erios@ipatimup.pt (E.R.); ssimoes@ipatimup.pt (M.S.-S.)

[2] Institute of Molecular Pathology and Immunology of the University of Porto (Ipatimup), 4200-135 Porto, Portugal; celoy@ipatimup.pt

[3] Research Coordination, National Institute of Cancer, Rio de Janeiro 20230-130, Brazil; anaclarafonseca_93@hotmail.com

[4] Medical Faculty, University of Porto, 4200-319 Porto, Portugal

[5] INEB—Instituto de Engenharia Biomédica, 4200-135, Porto, Portugal

[6] ICBAS—Instituto de Ciências Biomédicas Abel Salazar da Universidade do Porto, 4050-313 Porto, Portugal

[7] Department of Pathology, Hospital de S. João, 4200-319 Porto, Portugal

[8] Natural Sciences Department, Health and Humanities Institute, Fluminense Federal University, Rio de Janeiro 28880-000, Brazil

* Correspondence etelgimba@id.uff.br (E.R.P.G.), psoares@ipatimup.pt (P.S.). Tel.: +55-21-3207-6599 (E.R.P.G.); Tel.: +315-225513618 (P.S.)

Received: 29 August 2018; Accepted: 27 September 2018; Published: 30 September 2018

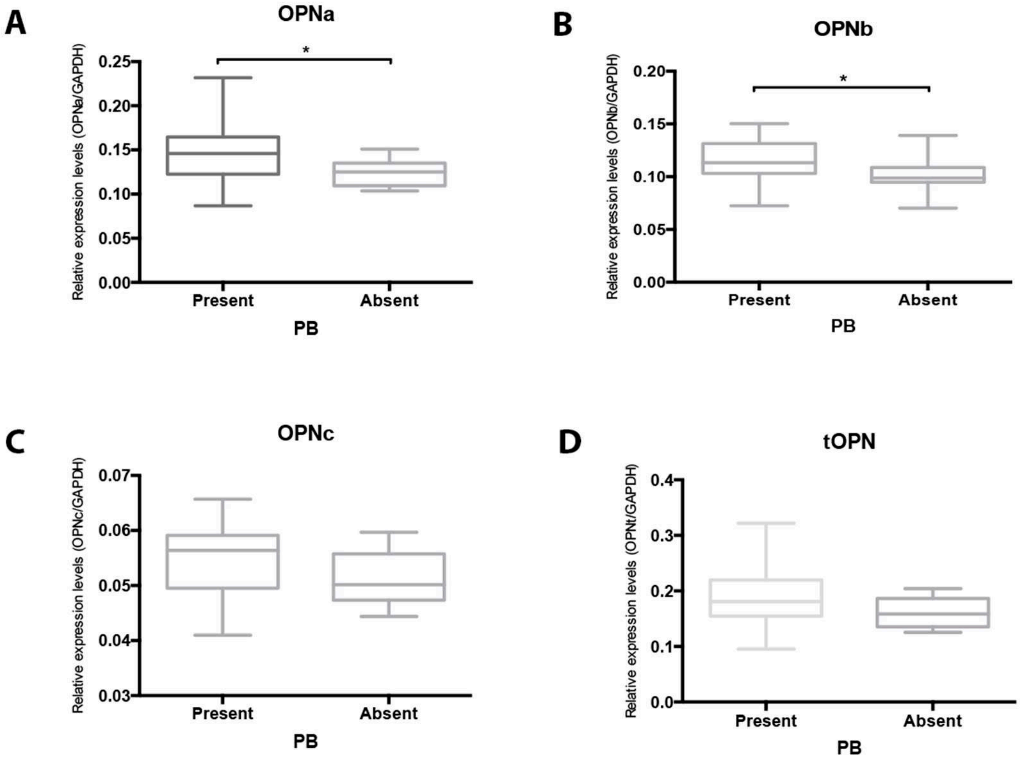

Figure S1. Expression levels of OPNa, OPNb, OPNc and tOPN transcripts in cPTC samples, concerning presence or absence of PB. (A) OPNa (B) OPNb (C) OPNc and (D) tOPN mRNA expression levels were measured by real <me PCR in the cPTC samples. * $p < 0.05$. Results are from at least two independent assays with triplicates.

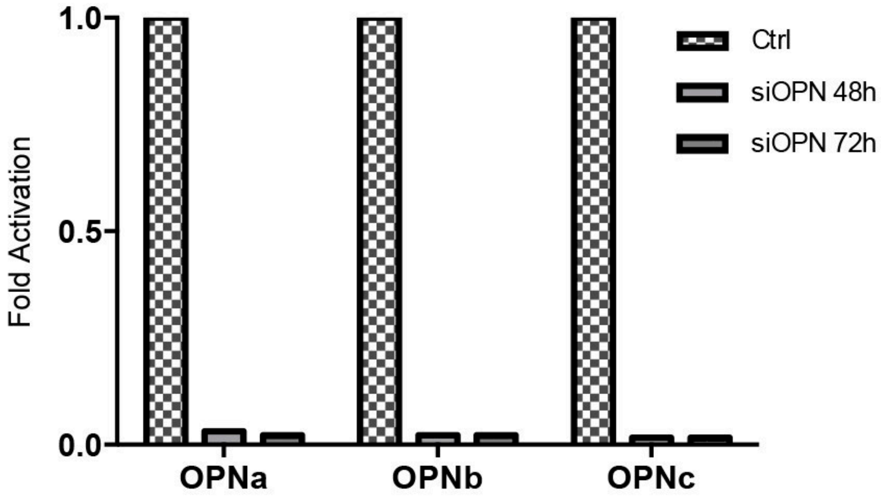

Figure S2. Expression levels of three OPN splice variants in response to total OPN silencing. OPNa, OPNb and OPNc mRNA expression levels have been measured by real time PCR in TPC1 thyroid cell line after 48 and 72 hours post total OPN (tOPN) silencing. Cells were treated with 100nM of siRNA for tOPN and 100nM of siRNA negative control (ctrl) during 48h and 72h.

Table S1. Staining score of tOPN IHC in cPTC samples.

OPN Staining Score*	n	%
0	19	39. 6
1	1	2. 1
2	7	14. 6
3	6	12. 5
4	2	4. 2
5	7	14. 6
6	5	10. 4
7	1	2. 1

* Staining intensity plus % of positive stained cells; osteopontin (OPN).

Table S2. Correlation between tOPN protein expression and the clinicopathological characteristics in cPTC.

Variable	N (%)	OPN Score Mean	p value
Sex *			
Male	6 (13)	1.50	0.34
Female	41 (87)	2.49	
Age (years) *			
< 45	26 (55)	2.15	0.48
≥ 45	20 (45)	2.65	
Stroma			
Absent	21 (43)	1.48	0.01
Present	27 (57)	3.04	
Tumor Size (cm) *			
< 2	17 (37)	2.94	0.22

≥ 2	29 (63)	2.07	
Extrathyroid Extension*			
Absent	24 (57)	1.79	0.09
Present	18 (43)	3.00	
Invasion (capsular and/or vascular) *			
Absent	15 (35)	1.93	0.49
Present	28 (65)	2.46	
Lymph Node Metastases *			
Absent	21 (60)	2.33	0.71
Present	14 (40)	2.64	
RET/PTC rearrangement *			
Absent	37 (84)	2.57	0.88
Present	7 (16)	2.43	
$BRAF^{V600E}$ mutation *			
Absent	22 (48)	2.64	0.62
Present	24 (52)	2.29	
RAS mutation *			
Absent	44 (96)	2.57	0.11
Present	2 (4)	0	

* Clinicopathological data was missing in some cases included in the series.

Table S3. Correlation between the presence of PB and the clinicopathological characteristics in cPTC.

	Psammoma Bodies [1]		
Total n = 45	Absent n = 21 (%)	Present n = 24 (%)	p value
Sex			
Male	2 (9.5)	2 (8.3)	NS (1.0)
Female	19 (90.5)	22 (91.7)	
Age (years)			
<45	7 (33.3)	18 (75)	0.005
≥45	14 (66.7)	6 (25)	
Stroma			
Absent	11 (52.4)	8 (33.3)	NS (0.1)
Present	10 (47.6)	16 (66.7)	
Tumor size (cm)			
<2	10 (47.6)	8 (34.8)	NS (0.3)
≥2	11 (52.4)	15 (65.2)	
Capsule			
Absent	9 (47.4)	14 (63.6)	NS (0.2)
Present	10 (52.6)	8 (36.4)	
Extrathyroid Extension			
Absent	10 (58.8)	13 (56.5)	NS (0.8)
Present	7 (41.2)	10 (43.5)	
Invasion (Vascular and/or capsular)			
Absent	9 (47.4)	4 (19)	NS (0.1)
Present	10 (52.6)	17 (81)	
Lymph node metastases			
Absent	11 (84.6)	9 (42.9)	0.030
Present	2 (15.4)	12 (57.1)	
RET/PTC translocation			
Absent	18 (90)	17 (77.3)	NS (0.4)
Present	2 (10)	5 (22.7)	
$BRAF^{V600E}$ mutation			
Absent	9 (42.9)	12 (52.2)	NS (0.5)
Present	12 (57.1)	11 (47.8)	
RAS mutation			
Absent	20 (95.2)	22 (95.7)	NS (1.0)

Present	1 (4.8)	1 (4.3)

Abbreviations: NS, not significant. Number in parentheses represent percentages within each category. Bold values indicate the result was statistically significant.

Table S4. Predictive factors for lymph node metastases in papillary thyroid carcinomas.

	Lymph Node Metastases (n = 34)				
	Presence (%)	Univariate Analysis		Multivariate Analysis	
		OR (95% CI)	P value	OR (95% CI)	P value
Total	14 (41)				
Sex					
Male	1 (7.1)	1 (Referent)			
Female	13 (92.9)	1.4 (0.8–25)	NS (0.8)		
Age, years					
< 45	11 (78.6)	4.4 (0.9–21)	0.05	3.1 (0.5–16)	NS (0.1)
≥ 45	3 (21.4)	1			
Stroma					
Absent	4 (28.6)	1			
Present	10 (71.4)	1.5 (0.3–6.5)	NS (0.5)		
Tumor size (cm)					
< 2	5 (35.7)	1			
≥ 2	9 (64.3)	1.3 (0.3–5.4)	NS (0.6)		
PB					
Absent	2 (14.3)	1			
Present	12 (85.7)	7.3 (1.2–41)	0.02	5.6 (0.9–34)	0.05
Capsule					
Absent	8 (61.5)	2.1 (0.5–8.7)	NS (0.2)		
Present	5 (38.5)	1			
Extrathyroid extension					
Absent	6 (46.2)	1			
Present	7 (53.8)	2.0 (0.4–8.3)	NS (0.3)		
Invasion (Vascular and/or Capsular)					
Absent	2 (14.3)	1			
Present	12 (85.7)	4.3 (0.7–25)	NS (0.09)		
$BRAF^{V600E}$ mutation					
Absent	8 (61.5)	1			
Present	5 (38.5)	2.1 (0.5–8.7)	NS (0.2)		

OR: odds ratio; CI: confidence interval; NS: non-significant; PB: psammoma bodie.

Table S5. Forward and reverse oligonucleotide sequences.

Gene	Oligonucleotide name	Sequence 5'-3'
OPNa	OPNa F	ATC TCC TAG CCC CAC AGA AT
	OPNa R	CAT CAG ACT GGT GAG AAT CAT C
OPNb	OPNb F	CTC CTA GCC CCA CAG ACC CT
	OPNb R	TAT CAC CTC GGC CAT CAT ATG
OPNc	OPNc F	CTG AGG AAA AGC AGA ATG
	OPNc R	AAT GGA GTC CTG GCT GT
tOPN	tOPN F	CCA ACG AAA GCC ATG ACC AC
	tOPN R	CTG TGG GGA CAA CTG GAG TG
Osteocalcin	OC F	GGC GCT ACC TGT ATC AAT GG
	OC R	TCA GCC AAC TCG TCA CAG TC
Collagen Type I	Col 1A1 F	CCC CTG GAA AGA ATG GAG AT
	Col 1A1 R	AAT CCT CGA GCA CCC TGA G
GAPDH	GAPDH F	TCC CAT CAC CAT CTT TCA GGA GCA
	GAPDH R	TTC TAC ATG GTG GTG AAG ACG CCA
